# Temporal and geographical variation in low carbon inhaler dispensing in England, 2016 to 2021: an ecological study

**DOI:** 10.1177/01410768221133566

**Published:** 2022-11-16

**Authors:** Jianghan Tian, Anita McGrogan, Matthew D Jones

**Affiliations:** 1 School of Chemistry, University of Bristol, Bristol, BS8 1TS, UK; 2 Department of Life Sciences, University of Bath, Bath, BA2 7AY, UK

**Keywords:** Asthma, respiratory system, pulmonary emphysema,

## Abstract

**Objectives:**

In 2019–2020, four national recommendations were published in the United Kingdom to encourage use of low carbon inhalers. This study aimed to investigate whether these were associated with a change in primary care dispensing in England and to explore associations between geographical variation and clinical commissioning group (CCG) characteristics.

**Design:**

Ecological study using aggregated publicly available data.

**Setting:**

All CCGs in England (March 2016 to February 2021).

**Participants:**

not applicable

**Main outcome measures:**

Percentage of low carbon inhalers dispensed.

**Results:**

The percentage of low carbon inhalers dispensed was 26.3% in 2020–2021 (of 8.8 million inhalers). This decreased over the study period for short-acting beta-agonist (SABA), inhaled corticosteroid (ICS) and ICS+long-acting beta-agonist (LABA) inhalers. The same trend was seen for LABA and ICS+LABA+long-acting muscarinic antagonist inhalers from 2019. The SABA and ICS classes were less often dispensed as low carbon inhalers (⁓6% versus 35–45%). Interrupted time series analyses found slight increases in low carbon inhaler percentage in the SABA, LABA and ICS classes after April 2019, which were soon erased by the long-term trend. There was also geographical variation, with the north-west, Birmingham and London consistently dispensing more low carbon inhalers. The presence of advice on climate change in CCG formularies/guidelines, the prevalence of asthma and population age profile were associated with significant variation in low carbon inhaler percentage for some classes.

**Conclusions:**

The percentage of low carbon inhalers dispensed in England remains low and continues to decrease. Greater use of low carbon inhalers is achievable, but is more likely with locally implemented initiatives.

## Introduction

To face the challenge of climate change, the UK government has committed to achieving net zero greenhouse gas emissions by 2050. This will require the National Health Service (NHS) in England to reduce its emissions, as they are currently equivalent to 4% of England’s total carbon footprint.^1^ Importantly, hydrofluoroalkane propellants released from pressurised metered dose inhalers (pMDIs) are powerful greenhouse gases and contribute 3% of the NHS’s carbon footprint.^1^ Typical pMDIs release 9–36 kg carbon dioxide equivalent (CO_2_e) per inhaler, whereas propellant-free dry powder inhalers (DPIs) have an estimated carbon footprint of <6 kg CO_2_e per inhaler.^2^ Similarly, life cycle analysis has estimated that pMDIs using current HFC-134a and HFC-227ea propellants have a global warming potential of 263 and 697 g** **CO_2_e per dose respectively, whereas DPIs have a global warming potential of just 9 g** **CO_2_e per dose.^3^ However, DPIs were assessed as having a larger environmental impact than pMDIs in 8 of 14 other categories, and potential future use of HFC-152a as a pMDI propellant would produce just 20 g** **CO_2_e per dose.^3^ In addition, evidence suggests that there is no difference in the efficacy of pMDIs and DPIs in asthma and chronic obstructive pulmonary disease (COPD) when patients have good inhaler technique.^4^ Consequently, the 2019 NHS long-term plan proposed increased use of lower carbon inhalers in order to reduce use of pMDIs.^5^

In line with this high-level policy, in the 12-month period beginning April 2019, four documents were published by respected professional bodies in the UK with the aim of increasing use of low carbon inhalers such as DPIs. In April 2019, the National Institute for Health and Care Excellence (NICE) published a patient decision aid highlighting the carbon footprint of different types of inhaler in asthma.^6^ Three months later, an update to the British Guideline on the Management of Asthma included a new section on the environmental impact of pMDIs and recommended ‘that inhalers with low global-warming potential should be used when they are likely to be equally effective’.^7^ In February 2020, the Primary Care Respiratory Society’s position statement on this issue recommended a multifaceted approach, including improvements in diagnosis and disease management, increased inhaler recycling and switching from pMDIs to propellant-free inhalers ‘where the change is clinically appropriate, safe and acceptable to patients’.^8^ This document also highlighted the importance of individual patients’ clinical needs remaining the primary focus for clinicians and did not support mass switching between types of inhalers. Similarly, in March 2020, the British Thoracic Society published a position statement on the environment and lung health that recommended the prioritisation of DPIs, switching from pMDIs to DPIs where patients can use their new inhaler safely, optimisation of inhaler technique and increased inhaler recycling.^9^ However, other authors have concerns about some of these approaches, including the risk of destabilising disease control when switching between inhalers, the wider environmental impact of DPIs and the advantages of pMDIs for certain patient groups, such as the very young and old.^10,11^

There are many barriers to reducing pMDI use that may not have been addressed by the publication of such documents. For example, some DPIs have a greater upfront cost than equivalent pMDIs,^2^ a pMDI with spacer is preferred for young children^7^ and some elderly people may be unable to generate sufficient inspiratory flow to use a DPI.^12^ Some authors have suggested that marketing strategies and prescriber and patient preferences also influence choices between pMDIs and DPIs.^13^ It has also been suggested that advice on the environmental impact of inhalers could be expressed more explicitly and so some clinicians may not be aware of this topic, especially if local policies and guidelines have not been updated.^14^ Finally, ethical and safe switching of an established pMDI to a DPI requires a face-to-face consultation to obtain the patient’s informed consent, identify a DPI that they can use appropriately and provide training.^14^ The capacity to provide such consultations may also be a barrier to reducing pMDI use. The key influences on inhaler selection therefore include cost, healthcare professional knowledge of devices, the inhalation manoeuvre achieved, the patient’s ability to use their device correctly and their personal preferences.^15^ It is therefore uncertain whether the recent guidelines and position statements are sufficient to change prescribing behaviour, especially as the most recently published data on inhaler usage in the UK relate to 2017,^2^ so evaluation of the impact of such documents has been recommended.^14^

The aim of this study, therefore, was to investigate if recent recommendations are associated with a change in low carbon inhaler dispensing in primary care in England. Specific objectives were:

- To quantify temporal and geographical variation in the proportion of low carbon inhaler dispensing relative to total inhaler dispensing over the past five years;
- To explore the association between geographical variation and variables such as asthma and COPD prevalence, population age profile and the content of local guidelines.

## Methods

This ecological study used aggregated data from several publicly available data sources in England. Data related to primary care, as this is where most inhalers are prescribed.

### Dispensing data

Clinical commissioning group (CCG)-level dispensing data for England from March 2016 to February 2021 were obtained from OpenPrescribing.net.^16^ The monthly number of inhaler items dispensed and their cost (at the ‘product format’ level) were extracted for the five pharmacological classes of inhaler where both pMDIs and low carbon inhalers were available: short-acting beta-agonist (SABA), long-acting beta-agonist (LABA), inhaled corticosteroid (ICS), ICS plus LABA combination (ICS+LABA) and ICS+LABA plus long-acting muscarinic antagonist combination (ICS+LABA+LAMA) devices. Within these classes, pMDIs and low carbon inhalers (DPIs and soft mist inhalers) were identified (Table S1, supplemental material). Other pharmacological classes were excluded, as they did not include both pMDIs and low carbon inhalers. Items were defined as the number of times an inhaler was dispensed; the number of individual inhalers dispensed was not available.

### CCG characteristics data

CCG population age profiles (percentage aged under 15 and over 80 years) for 2019 were obtained from the Office for National Statistics^17^ (Table S2, supplemental material). CCG asthma and COPD prevalence (%), emergency hospital admissions (EHA; per 100,000 population), mortality rates (per 100,000 population) and adult smoking prevalence (%) were obtained from Public Health England^18^ (Table S2, supplemental material). Local formularies and guidelines on CCG public websites in May 2021 were reviewed to record the presence or absence of advice on the carbon footprint of inhalers and the number of recommended pMDIs and low carbon inhalers in each pharmacological class (Tables S3 and S4, supplemental material). A small amount of CCG data were unavailable from their respective data sources (Table S5, supplemental material).

### Statistical analyses

Statistical analyses were performed using RStudio Version 1.4.1717. To control for changes in the volume of inhaler dispensing, the key outcome measure used in this study is the percentage of low carbon inhalers dispensed, defined as the number of low carbon inhaler items dispensed relative to the total number of pMDI and low carbon inhaler items. In addition, the average cost for pMDI and low carbon inhaler items was defined as the total cost of these items divided by the total number of items. Interrupted time series (ITS) analysis with segmented regression was used to investigate the temporal variation in low carbon inhaler percentage for all the five classes of inhalers.^19^ To fit the data, Poisson regression models were used for SABA, ICS and ICS+LABA inhalers and polynomial models for LABA and ICS+LABA+LAMA inhalers. Data for March 2020 were excluded since they were affected by the initial COVID-19 outbreak and were therefore outlying (Figure S1, supplemental material).

The consistency of CCG low carbon inhaler percentage between the five pharmacological classes was assessed by calculating Cronbach’s alpha for total dispensing from March 2020 to February 2021. Multivariable regression was used to investigate the geographical variation for all five classes of inhalers, each totalled over the 12-month period from March 2020 to February 2021. Dependent variables were low carbon inhaler percentage for LABA and ICS+LABA+LAMA classes, and the natural logarithm of low carbon inhaler percentage for SABA, ICS and ICS+LABA classes, for a better fit of the model and reduced multicollinearity. Univariable Pearson’s correlation analyses were used to identify independent variables for inclusion in multivariable regression models; those with a coefficient of determination >0.1 for at least one class were included for all classes, along with those that were clinically useful. This led to the rejection of adult smoking prevalence and the number of recommended pMDIs in CCG formularies as independent variables. Therefore, multivariable regression models were constructed with the independent variables being the other CCG population and guideline characteristics described in Section ‘CCG characteristics data’.

## Results

### Temporal variation of low carbon inhaler dispensing

Table 1 summarises the dispensing and average cost of pMDIs and low carbon inhalers in the five pharmacological classes over the five-year study period. pMDI SABA inhalers were the most frequently dispensed and least expensive. The average cost of low carbon SABA inhalers was three times greater. The SABA and ICS classes were more frequently dispensed as pMDIs compared with other classes (low carbon inhaler percentage ⁓6% versus 35%–45%). The average cost of pMDI and low carbon inhalers was similar (within 3.5%) in the LABA, ICS+LABA and ICS+LABA+LAMA classes. ICS+LABA and ICS+LABA+LAMA inhalers had the greatest low carbon inhaler percentage (⁓45%). The total low carbon inhaler percentage across all five classes increased over the study period from 19.5% (first 12 months) to 26.3% (last 12 months).

**Table 1.** Summary of monthly dispensed items and average cost for five pharmacological classes of inhalers (March 2016 to February 2021).

	SABA n = 60 months		LABA n = 60 months		ICS n = 60 months		ICS+LABA n = 60 months		ICS+LABA+LAMA n = 24 months^a^	
	Mean (SD)	Min, Max	Mean (SD)	Min, Max	Mean (SD)	Min, Max	Mean (SD)	Min, Max	Mean (SD)	Min, Max
Low carbon inhaler items per month	103,817(9315)	84,425, 146,400	17,143(4200)	10,063, 24,712	33,815(3669)	27,860, 49,442	539,962(40,836)	452,193, 641,663	68,442(14,216)	40,432, 88,338
pMDI items per month	1,707,307(153,903)	1,446,499, 2,623,485	31,868(8636)	18,474, 48,731	521,269(51,019)	439,307, 829,346	639,876(64,780)	534,601, 893,171	84,114(21,639)	44,195, 116,642
Low carbon inhaler %	5.7%(0.3%)	5.3%, 6.3%	35.1%(0.7%)	33.6%, 36.1%	6.1%(0.4%)	5.5%, 6.9%	45.8%(3.5%)	40.3%, 51.5%	45.2%(1.5%)	42.9%, 47.8%
Low carbon inhaler cost (£/item)	£6.57	–	£33.44	–	£17.55	–	£35.90	–	£46.54	–
pMDI cost (£/item)	£2.17	–	£32.75	–	£10.83	–	£34.68	–	£45.15	–

^a^March 2019–February 2021, as most ICS + LABA + LAMA inhalers came to the market in late 2018.

SABA: short-acting beta-agonist; LABA: long-acting beta-agonist; ICS: inhaled corticosteroid; ICS+LABA: ICS plus LABA combination; ICS+LABA+LAMA: ICS+LABA plus long-acting muscarinic antagonist combination; pMDI: pressurised metered dose inhaler.

The monthly number of low carbon inhaler items showed a gradually decreasing trend for all classes except ICS+LABA+LAMA inhalers, where both pMDI and low carbon inhaler items increased quickly after these products came to the market in 2017–2018 (Figure S1, supplemental material). In the SABA and ICS classes, pMDI items were relatively stable over the five years, whereas ICS+LABA pMDI items increased. LABA pMDIs and low carbon inhaler items both decreased over the five years. Dispensed items for all categories of inhalers peaked in March 2020, during the initial outbreak of COVID-19 in the UK. In addition, there were small peaks in December and January each year for all types of inhalers.

**Figure 1.**
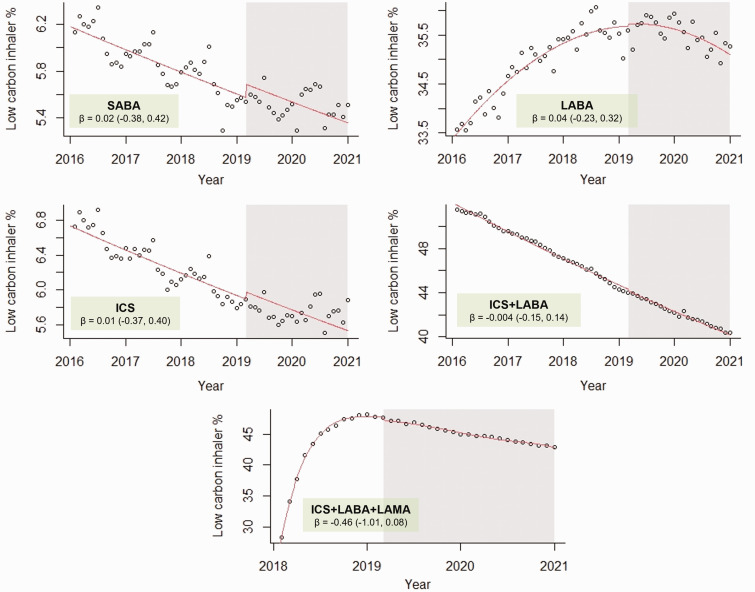
Interrupted time series analyses of monthly low carbon inhaler percentage dispensing in England from March 2016 to February 2021 for short-acting beta-agonist (SABA), long-acting beta-agonist (LABA), inhaled corticosteroid (ICS), ICS plus LABA combination (ICS+LABA) and ICS+LABA plus long-acting muscarinic antagonist combination (ICS+LABA+LAMA) devices. Black circles indicate the monthly low carbon inhaler percentage while the red line is the regression model of the interrupted time series analysis. The area of white background shows the pre-intervention period (before the NICE decision aid was released in April 2019); the area of grey background shows the post-intervention period (after the NICE decision aid was released). β is the regression coefficient describing the change in low carbon inhaler percentage following April 2019, with the 95% confidence interval shown in brackets.

Figure 1 shows the monthly low carbon inhaler percentage over the five-year study period for each pharmacological class, including the ITS analyses with the point of intervention in April 2019, when the NICE decision aid was released.^6^ Over the five years, there was a decreasing trend of similar relative magnitude for the SABA, ICS and ICS+LABA classes. Low carbon inhaler percentage increased for the LABA class over the five years, but showed a decreasing trend since 2019. ICS+LABA+LAMA low carbon inhaler percentage increased during their first year on the market (2018), but subsequently started to decrease. The SABA and ICS classes also showed seasonal variation in low carbon inhaler percentage, with peaks in the spring and summer months.

Small, non-significant increases in low carbon inhaler percentage were observed in the SABA, LABA and ICS classes after publication of the NICE decision aid in April 2019; however, this was soon erased by the long-term downward trend. Small, non-significant decreases were seen in the ICS+LABA and ICS+LABA+LAMA classes. Similar results were obtained with the point of intervention set in October 2019 and April 2020 (see Figures S2 and S3, supplemental material).

### Geographical variation in low carbon inhaler dispensing

**Figure 2.**
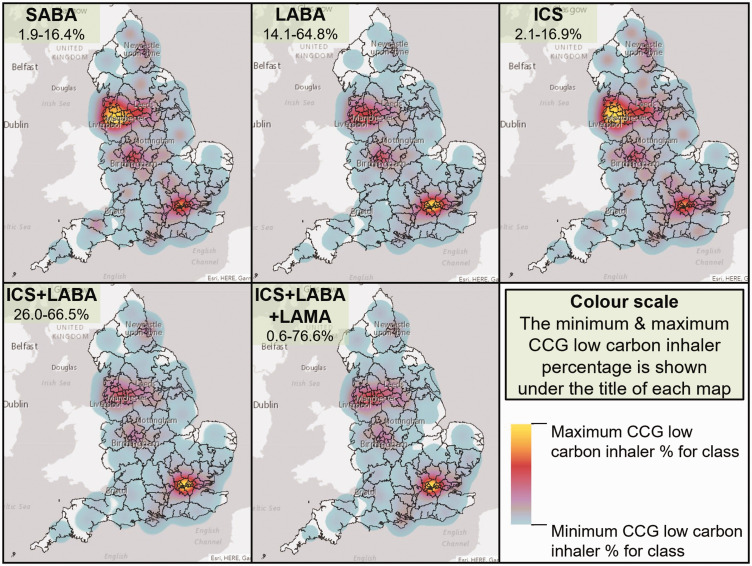
Low carbon inhaler percentage by clinical commissioning group from March 2020 to February 2021 for short-acting beta-agonist (SABA), long-acting beta-agonist (LABA), inhaled corticosteroid (ICS), ICS plus LABA combination (ICS+LABA) and ICS+LABA plus long-acting muscarinic antagonist combination (ICS+LABA+LAMA) inhalers. The numbers under the title of each map are the minimum and maximum CCG low carbon inhaler percentage for that class.

Figure 2 illustrates the geographical variation in low carbon inhaler dispensing by CCG. There were several areas with consistently higher low carbon inhaler percentage: for SABA and ICS devices the north-west was highest, while for LABA, ICS+LABA and ICS+LABA+LAMA inhalers the London area was highest. The Birmingham area was also consistently higher than many other areas. Cronbach's alpha was 0.40, suggesting there was little consistency between CCG’s low carbon inhaler percentages for the five pharmacological classes.

There were several geographical clusters of CCGs that provided advice on the carbon footprint of inhalers in their formularies of guidelines, such as in the east of London and the north west of England (Figure S4, supplemental material). A similar distribution of asthma and COPD prevalence was observed (Figure S5, supplemental material).

### Factors associated with geographical variation

Table 2 presents the results of the multivariable regression models of the variation in low carbon inhaler percentage between CCGs. For the SABA and ICS classes, the presence of advice on climate change in CCG formularies or guidelines was associated with significant increases in low carbon inhaler percentage of 26% and 28%, respectively, compared to those CCGs that did not provide this information (SABA: from 5.7% to 7.2%; ICS: from 6.1% to 7.8%). However, this association was not seen for the LABA, ICS+LABA and ICS+LABA+LAMA classes. The prevalence of asthma was associated with significant variation in low carbon inhaler percentage for the SABA and ICS classes, with a 30% increase in low carbon inhaler percentage associated with each 1% increase in asthma prevalence. The percentage of CCG population aged under 15 years was negatively associated with low carbon inhaler percentage for the ICS and ICS+LABA classes, with a 7% and 4% decrease in low carbon inhaler percentage for each 1% increase in the percentage of population aged under 15 years, respectively. Although some coefficients related to emergency hospital admissions and mortality rates were statistically significant, their absolute values were very small and clinically non-significant.

**Table 2.** Multivariable regression models examining the association between the low carbon inhaler dispensing and various CCG characteristics for five pharmacological classes.

Variable	SABA^a^	LABA	ICS^a^	ICS+LABA^a^	ICS+LABA+LAMA
	Model coefficient (95% CI)	Model coefficient (95% CI)	Model coefficient (95% CI)	Model coefficient (95% CI)	Model coefficient (95% CI)
Formularies and guidelines^b^	0.23*(0.01, 0.45)	−2.69(−7.69, 2.31)	0.25**(0.08, 0.43)	−0.07(−0.16, 0.02)	−0.003(−7.54, 7.53)
*Number of low carbon inhaler dispensing options in CCG local formulary*					
For asthma	−0.01(−0.17, 0.15)	0.80(−2.65, 4.25)	0.03(−0.02, 0.07)	0.01*(0.002, 0.03)	−7.18(−37.94, 23.58)
For COPD	−0.03(−0.23, 0.16)	−0.62(−2.02, 0.77)	–^c^	0.002(−0.02, 0.02)	−5.17(−10.76, 0.43)
*CCG disease prevalence(%)*					
Asthma	0.26*(0.06, 0.46)	−1.79(−6.94, 3.35)	0.26**(0.08, 0.45)	−0.10*(−0.19, −0.01)	−1.62(−8.98, 5.74)
COPD	−0.27(−0.69, 0.15)	8.55(−1.34, 18.44)	−0.14(−0.52, 0.25)	0.14(−0.04, 0.32)	1.28(−13.34, 15.91)
*Percentage of CCG population in specific age groups*					
<15 years	−0.04(−0.13, 0.04)	−0.09(−1.93, 1.75)	−0.07*(−0.14, −0.002)	−0.04*(−0.08, −0.01)	–^d^
>80 years	0.05(−0.14, 0.23)	−3.17(−7.62, 1.29)	−0.01(−0.18, 0.16)	−0.07(−0.15, 0.01)	−0.36(−6.35, 5.63)
*CCG emergency hospital admissions for adults (per 100,000 population)*					
For asthma	4.50E−6(−2.97E−5, 3.87E−5)	0.001*(0.0002, 0.002)	−9.30E−6(−4.13E−5, 2.27E−5)	3.06E−5***(1.60E−5, 4.52E−5)	0.0003(−0.0009, 0.002)
For COPD	−6.12E−6(−2.72E−5, 1.49E−5)	−0.0003(−0.0008, 0.0003)	−1.28E−5(−3.21E−5, 6.50E−6)	−8.09E−6(−1.75E−5, 1.36E−6)	0.0004(−0.0004, 0.001)
*CCG mortality rate (per 100,000 population)*					
From asthma	−0.0003(−0.002, 0.0009)	0.02(−0.009, 0.05)	−0.0001(−0.001, 0.0009)	−0.0006*(−0.001, −5.06E−5)	−0.06**(−0.10, −0.01)
From COPD	0.0001(−2.12E−5, 0.0003)	−0.003(−0.007, 0.0006)	0.0001(−1.77E−6, 0.0003)	−2.21E−5(−7.18E−5, 6.74E−5)	−0.003(−0.008, 0.003)

*p** **<** **0.05; **p** **<** **0.01; ***p** **<** **0.001.

^a^Model used natural logarithm of low carbon inhaler percentage, for a better fit.

^b^The presence of advice on the carbon footprint of inhalers in CCG formulary or guidelines.

^c^Variable not included in the model, as no ICS inhalers are licenced for COPD.

^d^Variable not included in the model, ICS+LAMA+LABA inhalers are not licensed for use in children.

SABA: short-acting beta-agonist; LABA: long-acting beta-agonist; ICS: inhaled corticosteroid; ICS+LABA: ICS and LABA combination; ICS+LABA+LAMA: ICS+LABA and long-acting muscarinic antagonist combination; CCG: clinical commissioning group; COPD: chronic obstructive pulmonary disease.

## Discussion

The overall use of low carbon inhalers in this study was low (26.3%), which is similar to studies examining the years 2002–2008 in the UK^13^ and 2017 in England,^15^ when approximately 70% of inhalers sold or dispensed were pMDIs. Even for the pharmacological classes with the highest proportion of low carbon inhalers, over half of items dispensed during the study period were pMDIs. This is the first study to report temporal trends in low carbon inhaler use. Although the overall low carbon inhaler percentage increased from 2016 to 2021, this was driven by the introduction of ICS+LABA+LAMA inhalers in 2018. All classes of inhaler showed a trend of decreasing low carbon inhaler use since 2019, and for the SABA, ICS and ICS+LABA classes this trend was seen throughout the five-year study period. This has occurred despite the publication of national guidelines and policies that promote the use of low carbon inhalers,^6–9^ which were not followed by significant changes in the dispensing of low carbon inhalers.

There were also large differences in low carbon inhaler dispensing between classes. The lowest proportion was for SABA and ICS inhalers, which were two of the three most commonly dispensed classes. Previous studies have not considered variation between such specific classes of inhaler, but a study based on English dispensing data in 2017 found similar proportions of pMDI dispensing for both the SABA class (94%) and ‘devices that contained an ICS’ (62%; the equivalent proportion for the five years of this study for the ICS, ICS+LABA and ICS+LABA+LAMA classes combined was 66%).^15^ This might be related to the higher cost of low carbon inhalers in the SABA and ICS classes,^2^ whereas costs for low carbon inhalers and pMDIs were similar in the other three classes. In addition, national guidelines recommend use of a SABA pMDI with spacer during mild–moderate asthma attacks.^7^

There was considerable geographical variation in low carbon inhaler dispensing for each class, with evidence of similar prescribing practices in neighbouring CCGs. This has not been previously investigated for low carbon inhalers, but regional variation has previously been observed in other aspects of the treatment of asthma and COPD in the UK^20,21^ and other countries.^22^ The presence of advice on climate change in CCG formularies or guidelines was associated with small increases in the proportion of low carbon inhaler dispensing for the two classes with the lowest overall percentage (SABA and ICS). This suggests that local initiatives may be important in achieving this NHS long-term plan ambition, as has also been found in studies of both respiratory and non-respiratory diseases.^20,23,24^ A higher proportion of the CCG population aged under 15 years was associated with a lower proportion of ICS and ICS+LABA low carbon inhaler dispensing, which may be related to the national guideline recommendation to use a pMDI and spacer for young children.^7^ However, the same guidelines also found high-quality evidence that for the delivery of beta-agonists and ICS in stable asthma there is similar safety and efficacy data for both pMDIs and DPIs in people aged over five years, although as this evidence is 20 years old it is not based on more recent DPI designs.

### Strengths and limitations

The first strength of this study is its novel exploration of temporal and geographical variation in the dispensing of lower carbon inhalers in five specific pharmacological classes. The second strength is the use of detailed dispensing data covering the whole of England over a five-year period, thus reducing the risk of bias from sampling or reliance on surrogate measures. An important limitation is that the available data present the number of dispensed items instead of the number of inhalers, but there is no evidence to suggest that the number of inhalers per item varies between pMDIs and low carbon inhalers, so this limitation is unlikely to have affected the findings. The use of aggregated rather than individual-level dispensing and population characteristics data limited the ability to explore the influence of individual patient factors on low carbon inhaler use. In addition, the study period included the first year of the COVID-19 pandemic in the UK, which caused disruption to prescribing patterns and longer-term NHS projects, such as those intended to reduce pMDI use. Finally, causal inferences cannot be drawn from these data, so findings should be interpreted with caution.

### Implications

There are several significant implications of this study. First, it is clear that the national initiatives introduced up until the end of March 2021 were insufficient to achieve the ambition of the NHS’s long-term plan to increase use of low carbon inhalers. However, local initiatives by CCGs may have had some influence. Although this policy has been contested by some clinicians, further initiatives will be required if it is still the aim of the NHS to achieve this objective. This study suggests that such initiatives should include at least some elements that are implemented locally and that focusing on the ICS class might be an initial priority, as it is the third most frequently dispensed class and currently has a very low proportion of low carbon inhalers. While SABA inhalers are the most frequently dispensed and have the lowest proportion of low carbon inhalers, national guidelines currently recommend use of SABA pMDIs in acute asthma, so initiatives focused on this class may be less appropriate. Such approaches will need to overcome the barrier of the greater financial cost of SABA and ICS low carbon inhalers. A focus on the use of low carbon inhalers for older children prescribed ICS and ICS+LABA inhalers may also be beneficial, while still considering that over half of LABA, ICS+LABA and ICS+LABA+LAMA inhalers currently used in England are pMDIs. Further research is required to develop and validate interventions based on these recommendations.

From October 2021, NHS England and Improvement’s Impact and Investment Fund included a series of financial incentives for primary care networks to encourage increased use of low carbon inhalers.^25^ As this initiative will be locally implemented, aimed at non-SABA inhalers and addresses financial barriers, it is partially aligned with the findings of this research and may prove to be more successful than the approaches employed so far. However, it should be noted that this scheme was suspended in December 2021 in order to accelerate delivery of COVID-19 booster vaccinations.^25^

It is essential that all initiatives in this area require individual patients’ informed consent and that low carbon inhalers are only used when clinically appropriate, safe and acceptable to patients, whose individual needs must remain paramount.^8,11^ Whenever a new low carbon inhaler is started, whether as a new treatment or as a replacement for a pMDI, patients require a face-to-face consultation to provide training and ensure that they can use their new device correctly.^14^ As well as focusing on use of low carbon inhalers, further work is also required to ensure that the management of all patients with respiratory diseases is in line with current best practice,^8,11^ as this will benefit both individual patients and potentially the environment (through reduced consumption of resources to treat acute exacerbations). Finally, initiatives are also required to reduce the use of brands of pMDI with the highest carbon footprint and increase recycling of both pMDIs and DPIs, which may help to offset some of the other environmental impacts of these devices.^3,8,10,11^

## Conclusions

Despite the publication of numerous national policies and guidelines, the proportion of low carbon inhalers dispensed in England between 2016 and 2021 decreased for the SABA, ICS and ICS+LABA classes. The same trend was seen for the LABA and ICS+LABA+LAMA classes from 2019 onwards. The considerable variation between pharmacological classes and CCGs suggests that greater use of low carbon inhalers is achievable, but is more likely to be achieved with locally implemented initiatives focused on key barriers, classes of inhalers and types of patients.

## Data availability statement

Data are available in a public, open access repository. All data created during this research are openly available from the University of Bath Research Data Archive at https://doi.org/10.15125/BATH-01142

## Supplemental Material

sj-pdf-1-jrs-10.1177_01410768221133566 - Supplemental material for Temporal and geographical variation in low carbon inhaler dispensing in England, 2016 to 2021: an ecological study Click here for additional data file. Supplemental material, sj-pdf-1-jrs-10.1177_01410768221133566 for Temporal and geographical variation in low carbon inhaler dispensing in England, 2016 to 2021: an ecological study by Jianghan Tian, Anita McGrogan, Matthew D Jones in Journal of the Royal Society of Medicine

## Declarations

### Guarantor

MDJ.

### Provenance

Not commissioned; peer reviewed by Ervin Mingomataj and Julie Morris.

### ORCID iDs

Jianghan Tian https://orcid.org/0000-0002-5137-3363

Matthew D Jones https://orcid.org/0000-0002-2617-4098
