# Supplementary material for: Temporal and geographical variation in low carbon inhaler dispensing in England, 2016 to 2021: an ecological study
Source: J R Soc Med. 2022 Nov 16;116(2):65–75. doi: 10.1177/01410768221133566 (PMC9944236; doi:10.1177/01410768221133566)

Journal of the Royal Society of Medicine

Temporal and geographical variation in low carbon inhaler dispensing in England 2016 to 2021: an ecological study

Supplemental Material

Jianghan Tian[1], Anita McGrogan[2], Matthew D. Jones[2*]

[1] School of Chemistry, University of Bristol, Cantock's Close, BS8 1TS, Bristol, UK

[2] Department of Life Sciences, University of Bath, BA2 7AY, Bath, UK

*Corresponding author

Matthew D. Jones, Department of Life Sciences, University of Bath, Bath, UK, M.D.Jones@bath.ac.uk

Table S1. The categorisation of inhalers into pharmacological classes, pMDIs and low carbon inhalers.

Pharmacological class	Generic drugs included in pharmacological class	Product formats categorised as pressurised metered dose inhalers[1]	Product formats categorised as low carbon inhalers[1]
Short-acting beta-agonists (SABA)	Salbutamol<br>Terbutaline	Airomir<br>Airomir Autohaler<br>AirSalb<br>Salamol Easi-Breathe inhaler<br>Salamol inhaler<br>Salbutamol breath actuated inhaler<br>Salbutamol breath actuated inhaler CFC free<br>Salbutamol inhaler<br>Salbutamol inhaler CFC free<br>Ventolin Evohaler	Bricanyl Turbohaler<br>Easyhaler salbutamol sulfate<br>Pulvinal salbutamol<br>Salbulin Novolizer<br>Salbutamol dry powder inhalation cartridge<br>Salbutamol dry powder inhaler<br>Terbutaline dry powder inhaler<br>Ventolin Accuhaler
Long-acting beta-agonists (LABA)	Formoterol<br>Indacaterol<br>Olodaterol<br>Salmeterol	Atimos Modulite<br>Formoterol inhaler CFC free<br>Neovent<br>Salmeterol inhaler<br>Salmeterol inhaler CFC free<br>Serevent Evohaler<br>Serevent inhaler<br>Soltel CFC free<br>Vertine CFC free	Foradil<br>Formoterol dry powder inhaler<br>Formoterol Easyhaler<br>Formoterol inhalation powder capsules<br>Indacterol inhalation powder capsules<br>Olodaterol solution for inhalation<br>Onbrez<br>Oxis Turbohaler<br>Salmeterol dry powder inhaler<br>Salmeterol inhalation powder blisters<br>Serevent Accuhaler<br>Striverdi

Pharmacological class	Generic drugs included in pharmacological class	Product formats categorised as pressurised metered dose inhalers[1]	Product formats categorised as low carbon inhalers[1]
Inhaled corticosteroids (ICS)	Beclometasone<br>Budesonide<br>Ciclesonide<br>Fluticasone propionate<br>Mometasone	Aerobec Autohaler<br>Alvesco<br>Beclazone<br>Beclometasone breath actuated inhaler<br>Beclometasone breath actuated inhaler CFC free<br>Beclometasone inhaler<br>Beclometasone inhaler CFC free<br>Becotide<br>Budesonide inhaler<br>Budesonide inhaler CFC free<br>Ciclesonide inhaler CFC free<br>Clenil Modulite inhaler<br>Flixotide Evohaler<br>Fluticasone inhaler CFC free<br>Kelhale<br>Qvar Autohaler<br>Qvar inhaler<br>Qvar Easi-Breathe inhaler<br>Soprobec inhaler	Asmanex<br>Beclometasone dry powder inhaler<br>Becodisks<br>Budelin Novolizer<br>Budesonide dry powder inhaler<br>Budesonide dry powder inhalation cartridge<br>Easyhaler Beclometasone<br>Easyhaler Budesonide<br>Flixotide Accuhaler<br>Fluticasone propionate dry powder inhaler<br>Mometasone dry powder inhaler<br>Pulmicort Turbohaler

Pharmacological class	Generic drugs included in pharmacological class	Product formats categorised as pressurised metered dose inhalers[1]	Product formats categorised as low carbon inhalers[1]
ICS+LABA combination	Beclometasone and formoterol<br>Budesonide and formoterol<br>Fluticasone furoate and vilanterol<br>Fluticasone propionate and formoterol<br>Fluticasone propionate and salmeterol<br>Mometasone and indacaterol	AirFluSal<br>Aloflute<br>Beclometasone and formoterol inhaler CFC free<br>Budesonide and formoterol inhaler CFC free<br>Combisal<br>Fluticasone and formoterol breath actuated inhaler CFC free<br>Fluticasone and formoterol inhaler CFC free<br>Fluticasone and salmeterol inhaler CFC free<br>Flutiform<br>Flutiform K-haler<br>Fostair inhaler<br>Sereflo<br>Seretide Evohaler<br>Sirdupla<br>Symbicort pressurised inhaler	Aerivio<br>AirFluSal Forspiro dry powder inhaler<br>Beclometasone and formoterol dry powder inhaler<br>Budesonide and formoterol dry powder inhaler<br>DuoResp Spiromax<br>Fluticasone propionate and salmeterol dry powder inhaler<br>Fluticasone furoate and vilanterol<br>Fobumix Easyhaler<br>Fostair NEXThaler<br>Fusacomb Easyhaler<br>Indacaterol and mometasone powder capsules<br>Relvar Ellipta<br>Seretide Accuhaler<br>Stalpex<br>Symbicort Turbohaler
ICS+LABA+LAMA (long-acting muscarinic antagonist) combination	Beclometasone, formoterol and glycopyrronium<br>Budesonide, formoterol and glycopyrronium<br>Fluticasone furoate, vilanterol and umeclidinium<br>Mometasone, indacaterol and glycopyrronium	Trimbow[2]<br>Trixeo	Enerzair<br>Trelegy

[1]Unless stated, the listed descriptions include products of all doses that meet the description

[2]Trimbow NEXThaler was launched after the study period.

Table S2. Summary of clinical commissioning group population characteristics obtained from the Office for National Statistics and Public Health England

Variable	Mean (standard deviation)	Minimum value	Maximum value
Population under 15 years old (%)	18.1 (2.0)	14.5	25.9
Population over 80 years old (%)	5.0 (1.2)	1.6	8.6
Asthma prevalence (%)	6.6 (0.9)	3.5	8.3
COPD prevalence (%)	2.1 (0.6)	0.8	3.8
Asthma emergency hospital admissions per 100,000 population	111.6 (36.5)	46.4	248.1
COPD emergency hospital admissions per 100,000 population	275.6 (98.1)	135.2	557.3
Asthma mortality rate per 100,000 population	2.6 (0.9)	0.7	5.4
COPD mortality rate per 100,000 population	56.8 (17.4)	25.8	112.1
**Adult smoking prevalence (%)**	14.2 (2.9)	6.8	23.4

Table S3. Summary of clinical commissioning group (CCG) local guideline characteristics

**Number of CCGs with advice on the carbon footprint of inhalers present in local guidelines**	72
**Number of CCGs with advice on the carbon footprint of inhalers absent from local guidelines**	58

Table S4. Summary of clinical commissioning group local formulary characteristics

Variable	SABA	LABA	ICS	ICS+LABA	ICS+LABA+LAMA
Mean n**umber of** asthma **pMDIs** included (standard deviation)	1.7 (1.0)	0.1 (0.5)	3.3 (3.0)	4.3 (3.2)	0.0 (0.0)
Mean n**umber of** asthma **low carbon inhalers** included	1.5 (0.9)	0.1 (0.7)	2.2 (2.0)	6.7 (4.1)	0.0 (0.0)

(standard deviation)					
Mean number of COPD pMDIs included (standard deviation)	1.5 (0.8)	0.7 (1.0)	0.0 (0.0)	1.3 (0.9)	0.9 (0.3)
Mean number of COPD low carbon inhalers included (standard deviation)	1.1 (0.7)	1.2 (1.9)	0.0 (0.0)	3.2 (2.3)	1.1 (0.8)

Table S5. Quantity of missing data for variables related to Clinical Commissioning Group characteristics

Variable	Number of missing values	Missing data percentage
Asthma prevalence	0	0%
COPD prevalence	0	0%
Asthma emergency hospital admissions	18	13%
COPD emergency hospital admissions	18	13%
Asthma mortality rate	23	16%
COPD mortality rate	18	13%
Content of local formularies and guidelines[1]	5	4%

[1]Relevant formularies and guidelines not publicly available.

Figure S1. Temporal variation in the number of items dispensed in England over a 5-year period for (a) short-acting beta-agonist (SABA), (b) long-acting beta-agonist (LABA), (c) inhaled corticosteroids (ICS), (d) ICS plus LABA combination (ICS+LABA) and (e) ICS+LABA plus long-acting muscarinic antagonist combination (ICS+LABA+LAMA) devices. Blue lines represent the number of pMDIs, green lines represent the number of low carbon inhalers.

SABA – pMDIs

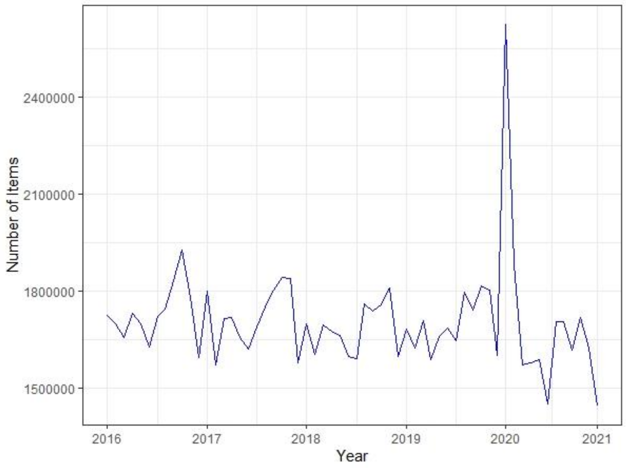

SABA – low carbon inhalers

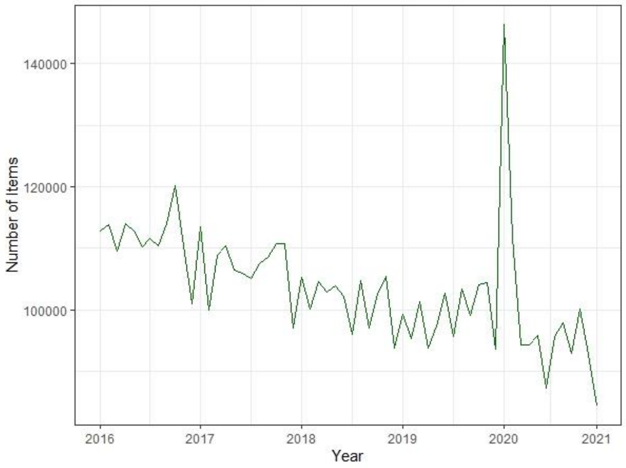

LABA

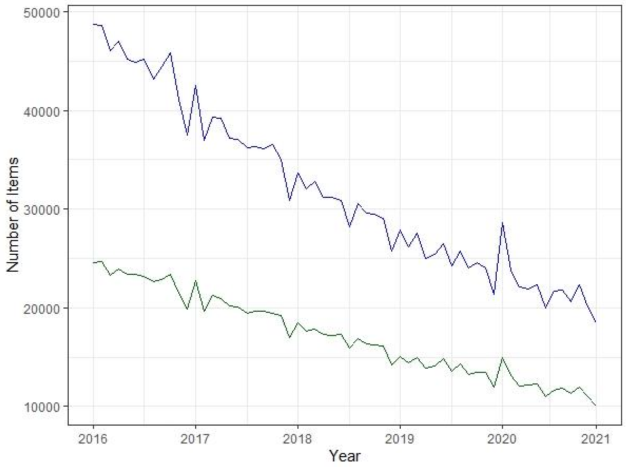

ICS – pMDIs

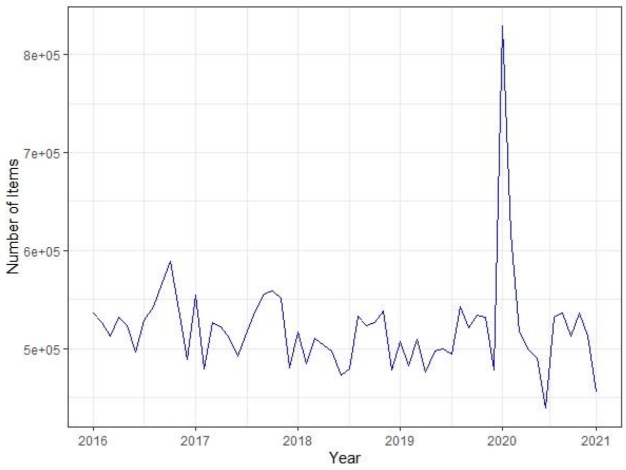

ICS – low carbon inhalers

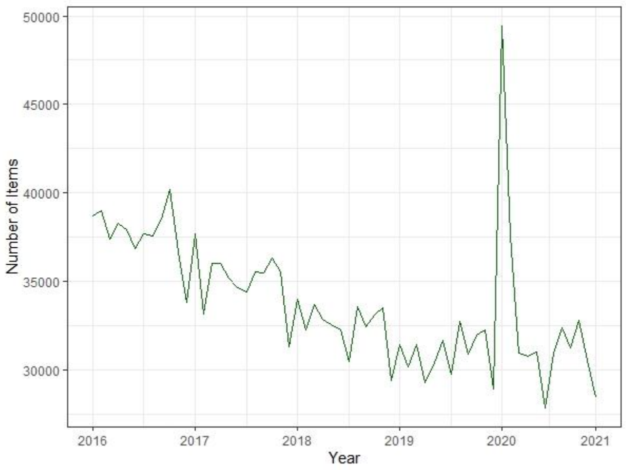

ICS+LABA

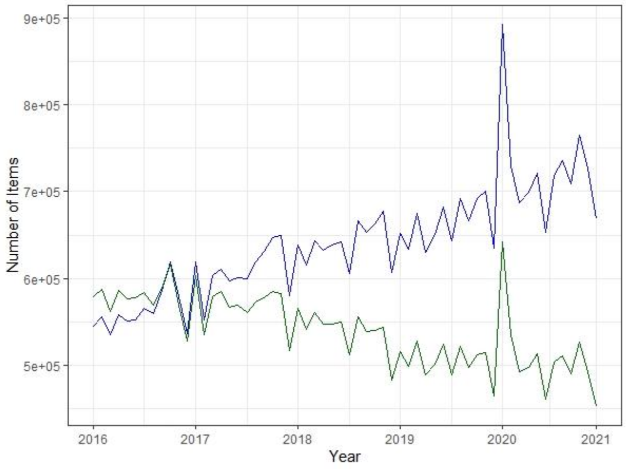

ICS+LABA+LAMA

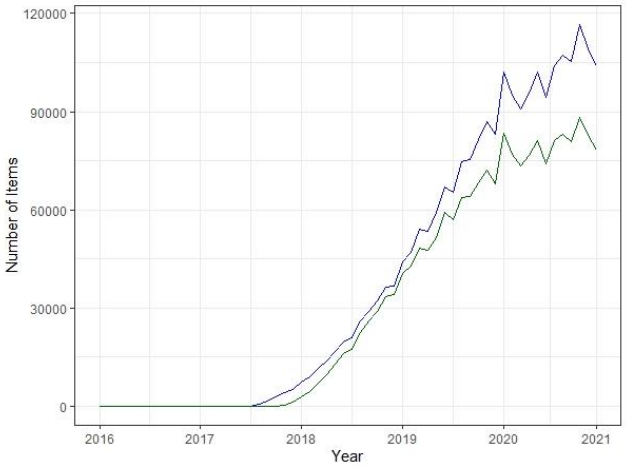

Figure S2. Interrupted time series analysis of the monthly low carbon inhaler percentage dispensing in England from March 2016 to February 2021 for short-acting beta-agonist (SABA), long-acting beta-agonist (LABA), inhaled corticosteroid (ICS), ICS plus LABA combination (ICS+LABA) and ICS+LABA plus long-acting muscarinic antagonist combination (ICS+LABA+LAMA) devices. Blue dotted line represents intervention point (October 2019, six months after NICE decision aid published).

SABA

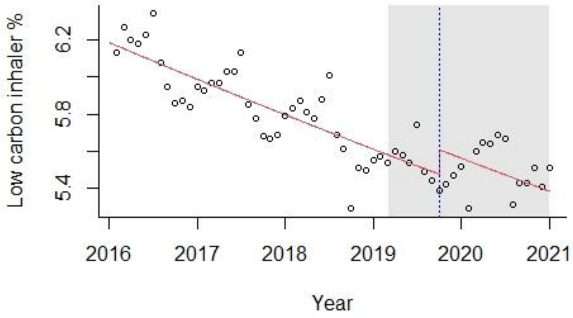

LABA

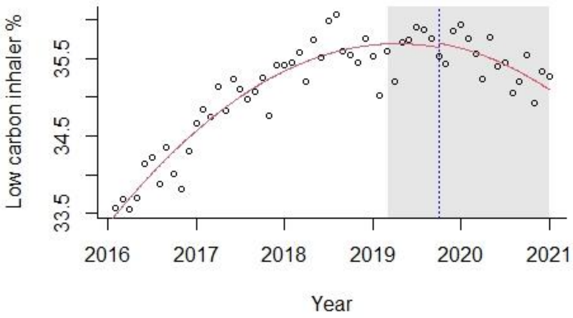

ICS

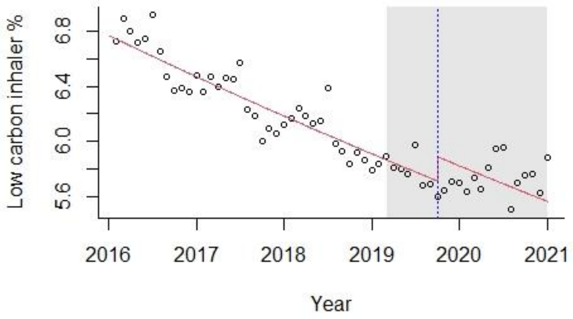

ICS+LABA

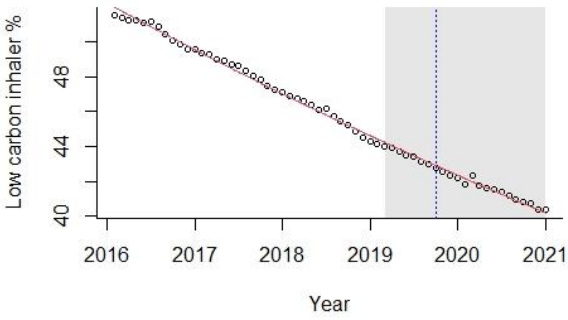

ICS+LABA+LAMA

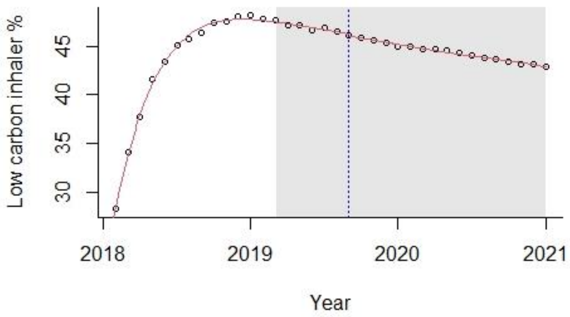

Figure S3. Interrupted time series analysis of the monthly low carbon inhaler percentage dispensing in England from March 2016 to February 2021 for short-acting beta-agonist (SABA), long-acting beta-agonist (LABA), inhaled corticosteroid (ICS), ICS plus LABA combination (ICS+LABA) and ICS+LABA plus long-acting muscarinic antagonist combination (ICS+LABA+LAMA) devices. Blue dotted line represents intervention point (April 2020, twelve months after NICE decision aid published).

SABA

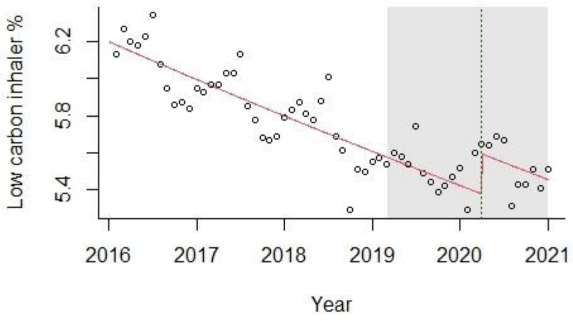

LABA

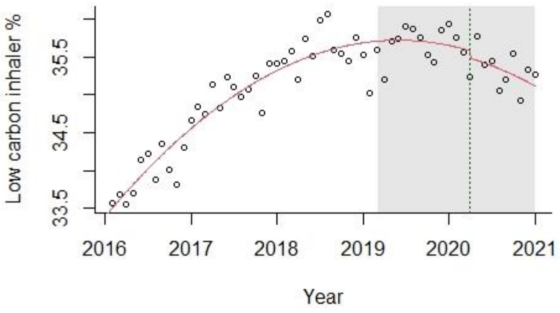

ICS

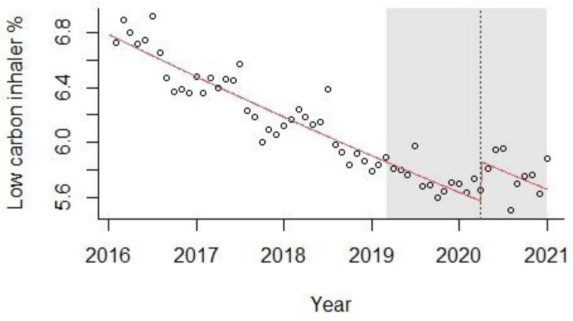

ICS+LABA

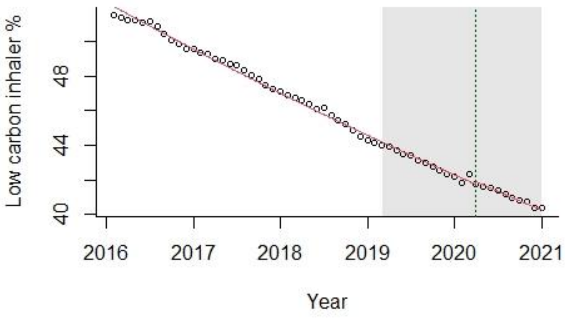

ICS+LABA+LAMA

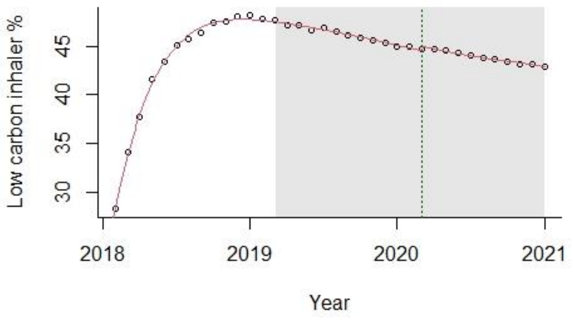

Figure S4. Geographical distribution of CCG formularies or guidelines that included advice on the carbon footprint of inhalers. Dark grey dots: CCG mentions climate change; light purple dots: CCG does not mention climate change.

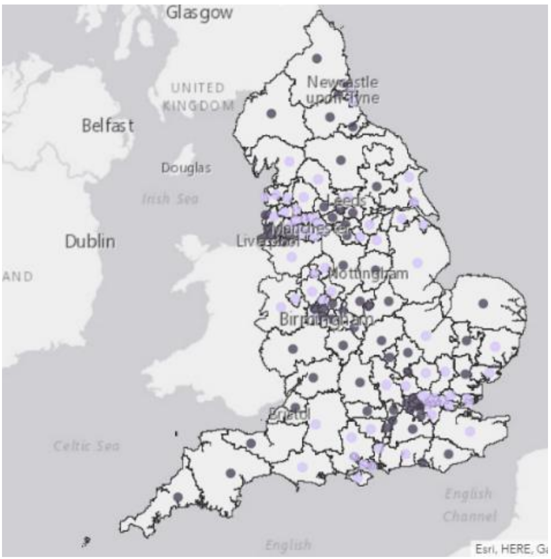

Figure S5. Heatmaps showing variation in clinical commissioning group QOF prevalence of asthma and COPD in England.

Asthma prevalence
Minimum: 3.5%; maximum: 8.3%

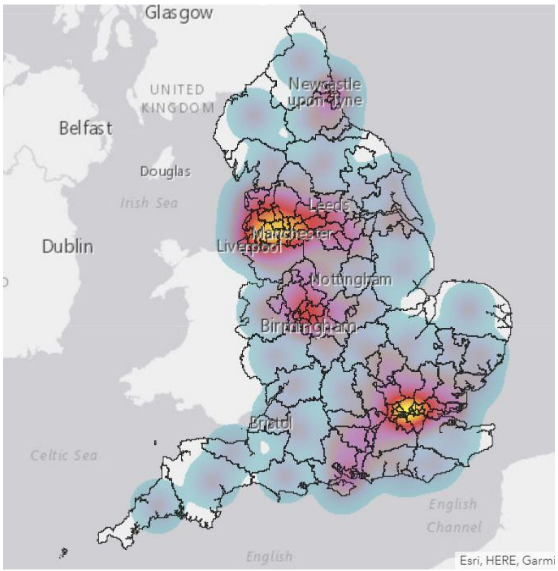

COPD prevalence
Minimum: 0.8%; maximum: 3.8%

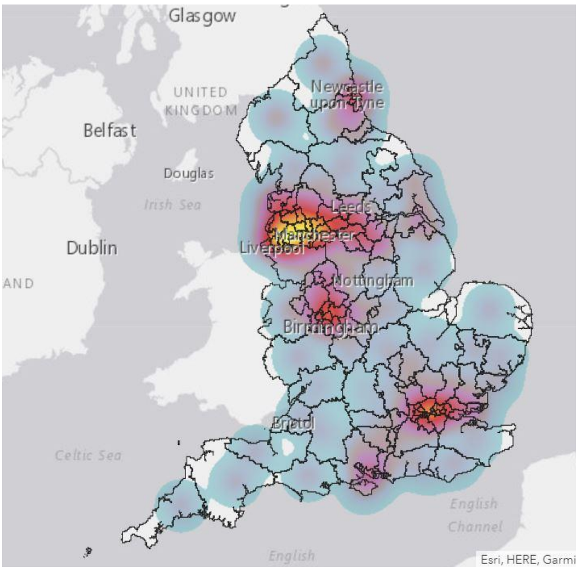

Colour scale
The minimum and maximum CCG prevalence is shown in the title of each map

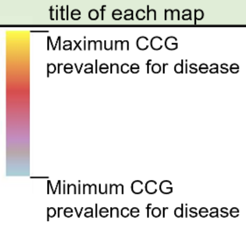

Supplement: sj-pdf-1-jrs-10.1177_01410768221133566 - Supplemental material for Temporal and geographical variation in low carbon inhaler dispensing in England, 2016 to 2021: an ecological study [file sj-pdf-1-jrs-10.1177_01410768221133566.pdf]
